# Practical Notes on Diagnosis and Treatment

**Published:** 1907-08-17

**Authors:** 


### Practical Notes on Diagnosis and Treatment.

### Lotion for Chapped Hands.

Caustic potash 8 grains, glycerine and rectified spirit, of each, 5 drachms, water 2 ozs. Wash the hands with warm water and then rub in the lotion freely.

### Sore Nipples.

The following ointments make suitable applications for this condition: Balsam of Peru ʒj., cold cream and lanoline, of each ʒiv. Or, tannic acid gr. xv., Friar's balsam ʒss., vaseline ℥j.

### Painful Rheumatic Joints.

As an application to the joints in cases of chronic rheumatism the following may be tried: Salicylic acid ʒj., oil of turpentine ʒj., lanoline and benzoated lard of each 1 oz.

### Operations and Pregnancy.

Never, if you can avoid it, perform an operation on a pregnant woman, for, however slight the operation may be, you will very likely produce abortion, from which very serious results may accrue.—*Mr. Christopher Heath.*

### Persisting Pyrexia.

In a case in which febrile temperatures persist for several weeks, and there are no physical signs adequate to explain them, enteric fever, tubercle, and septicæmia are all possible explanations. But, in addition, various diseases of the blood, and particularly pernicious anæmia, must not be forgotten.

### The Superficial Reflexes.

Excess of superficial reflex action is a common symptom in cases of compression of the cord, as distinguished from damage to the cord at a similar level by a primary morbid process within it. A great excess of superficial reflex action in the legs should always attract attention, and in some cases it is a trustworthy indication in the diagnosis between organic and functional disease.—*Sir Wm. Gowers.*

### Counter Irritation to the Spine.

The best method is the actual cautery. A Pacquelin's cautery, or even a good-sized copper wire, made red-hot, may be drawn along the skin for three or four inches on each side of the tips of the spinous processes. The application does not cause sufficient pain to demand a general anæsthetic, and the use of cocaine is inadvisable, as the benefit of counter-irritation is probably obtained through action on the nerves.

### Cardiac Failure.

Distension of the stomach or colon is a serious danger in cases where the heart is weakened by fever or other disease. Pressure of a distended stomach through the diaphragm on the right ventricle may readily, in such circumstances, cause fatal syncope. —*Dr. Sibson.*

### Blood Pressure.

A persistent systolic pressure of over 145 or 155 mm. of mercury recorded within an hour before a meal should be looked upon as suspicious, and in patients over 50 or 55 years of age should, if possible, be reduced by treatment, as it may gradually increase, and ultimately result in cerebral hæmorrhage or secondary cardiac disease.—*Dr. Geo. Oliver.*

### Rectal Troubles.

Patients who have trouble about the rectum should, as a rule, evacuate their bowels at night. Then they have eight to ten hours in the horizontal position, and the bowel has time to readjust itself by the time of rising. This is particularly applicable to those who have to leave home at a certain time every morning.—*Mr. Christopher Heath.*

### Dry Mouth.

For dryness of the buccal mucous membrane such as occurs in various febrile or other long illnesses, glycerine and water, in the proportion of one of the former to five of the latter, is very effective. A little lemon juice makes the mixture more palatable. Another useful preparation in similar circumstances is linseed tea with a little glycerine. For the troublesome dry mouth which accompanies certain forms of dyspepsia half-grain doses of calomel are useful; the calomel may be taken every night at bedtime.

### Treatment of Eclampsia.

On the first occurrence of an attack, or of the restlessness which frequently precedes an attack, chloroform should be administered and an enema containing 20 to 30 grains of chloral hydrate should be administered, the latter to be repeated in an hour's time if required. If possible the patient should be put into a hot bath. A plethoric person showing a good deal of cyanosis may be bled to 20 or 30 ozs. If chloroform and chloral fail you, morphine is a drug of great value; some authorities give as much as 2 to 3 grains in twelve hours. Morphine must be given very cautiously when the patient is passing only a small quantity of urine.—*Dr. G. F. Blacker.*

